# Growth performance, reproductive status, and chromosomal instability in triploid Nile tilapias

**DOI:** 10.1590/1984-3143-AR2023-0147

**Published:** 2024-05-24

**Authors:** Williane Ferreira Menezes, Érika Ramos Alvarenga, Rafael Henrique Nóbrega, Luiz Renato França, Marcelo Rezende Luz, Ludson Guimarães Manduca, Franklin Fernando Batista da Costa, Vinícius Monteiro Bezerra, Arthur Francisco de Araújo Fernandes, Eduardo Maldonado Turra

**Affiliations:** 1 Departamento de Zootecnia, Escola de Veterinária, Laboratório de Aquacultura, Universidade Federal de Minas Gerais, Belo Horizonte, MG, Brasil; 2 Departamento de Morfologia, Instituto de Biociências de Botucatu, Universidade Estadual Paulista Júlio de Mesquita Filho, Botucatu, SP, Brasil; 3 Cobb-Vantress, Inc., Siloam Springs, Arkansas, USA

**Keywords:** genomic instabilities, heat shock, Oreochromis niloticus, polyploidy, reproduction

## Abstract

Reproductive control is one of the biggest challenges in tilapia production and triploidy was developed as an alternative to sterilization. In general, polyploids present chromosomal instability but for triploid Nile tilapia it has yet to be reported. This study evaluated the chromosomal instability from juveniles to adulthood, growth performance and gonadal status of tilapia hatched from eggs submitted or not to heat shock for triploid induction. Nile tilapia oocytes were fertilized (1,476 oocytes), half of the eggs were subjected to a four-minute shock in 41 °C water four minutes after fertilization and the other half were not (Control group). The eggs were incubated (at 27°C) and 160 larvae from the treated group hatched and survived after yolk sac absorption. The determination of ploidy was performed by flow cytometry at 85^th^ (juveniles) and 301^st^ (adults) days of age post yolk sac absorption. At the time of the first cytometry analysis there were 73 surviving juveniles from the treated group, and only 14 were confirmed triploid. However, at the analysis of adult ploidy, one out of 8 surviving adult tilapias from the 14 confirmed triploid juveniles remained triploid. Gonadal histology showed that the non-remaining triploids continued to produce gametes. The growth performance of triploid tilapia was initially superior to that of diploid tilapia during the juvenile phase, but similar in adults. Once the chromosome sets are lost and the tilapias become diploid again, at least in tissues with a high proliferation rate, such as the hematopoietic tissue that was analyzed (and possibly in gonads), all possible advantages of triploids are probably lost. Thus, our results suggest that, due to genomic instabilities, the triploid generation of tilapia has low efficiency.

## Introduction

Although the introduction of tilapia in several countries has been a great success in the last three decades, there are discussions and concerns about possible environmental and biodiversity damage caused by this group of species (Teletchea and Fontaine, 2014). Reproductive control is one of the biggest challenges in tilapiculture and research has been conducted to develop techniques for tilapia spawning prevention. In this way, fish do not move energy to reproduction, only to growth. Lots of monosex fish or sterile individuals can be obtained by sexing, sexual manipulations, using sexual steroids or chromosomal manipulations such as polyploidy (Arai and Fujimoto, 2018; Baroiller and D’Cotta, 2018; Wang and Shen, 2018; Alvarenga et al., 2020; Costa e Silva et al., 2022). The effectiveness of sterility is also important for environmental sustainability because the escape of fertile fish represents a threat of genetic contamination of wild population stocks and possible ecological imbalance (Wong and Zohar, 2015).

Currently, the most used method on sexual control in commercial tilapia production is the use of monosex populations through the sexual inversion induction by hormonal treatment (Baroiller and D'Cotta, 2018; Costa e Silva et al., 2022). However, much is discussed about the sex steroids use in tilapia larvae, therefore, triploidy was considered as an alternative for reproductive control (Teletchea and Fontaine, 2014). The individual with three sets of chromosomes (3N), one paternal and two maternal (Budd et al., 2015) is a triploid. Triploidy can be induced by chromosomal manipulation, resulting in sterile individuals that circumvent the problem of early sexual maturity and unwanted reproduction (Pradeep et al., 2012).

Polyploidy increases the occurrence of spindle irregularities that can lead to disordered chromatids segregation, aneuploid cell production (abnormal chromosome numbers) and epigenetic instability (Borel et al., 2002). It is very common to study neoplastic cells to investigate the chromosomal aneuploid origin and karyotype instability (Reshmi et al., 2004; Silva et al., 2010), but it is relatively new to evaluate chromosomal instability in triploids and tetraploids fish. Aneuploidy can arise through two main mechanisms: cells can proceed through a tetraploid intermediate to a multipolar mitosis that creates a random chromosomal distribution, or they can proceed directly to aneuploidy through failure of a critical control of euploidy (Borel et al., 2002).

The lack of constancy in the number of chromosomes between polyploid suggests that polyploidy may not be a genetically stable combination (Comai, 2005). Alvarenga et al. (2020) observed a possible loss of chromosomes in Nile tilapia individuals during an attempt to induce tetraploidy, resulting in individuals aneuploid, mosaic or triploid. Chromosomal instability in tetraploid is considerably larger than in triploids, yet chromosomal triploid instability was 30 times higher than in diploids in *Saccharomyces cerevisiae* (Mayer and Aguilera, 1990). This instability in triploid and tetraploids was also demonstrated in Oysters (Sousa et al., 2016).

As far as we know, studies conducted with triploid Nile tilapia have evaluated ploidy status in the early stages of development (Hussain et al., 1991; Bramick et al., 1995; Byamungu et al., 2001; Pradeep et al., 2010, 2012, 2013, 2014) and the accompaniment of adult tilapias to verify chromosomal stability has not yet been reported. Thus, the objective of this study was to evaluate and to compare the chromosomal instability of triploid and diploid tilapia hatched from eggs subjected or not to heat shock, and to evaluate the growth and gonadal status of these animals.

## Methods

### Experimental installations and animals

Twenty-seven females (729 g ± 357) and 15 males (910 g ± 430) from the Nile tilapia broodstock (Chitralada lineage) of the NGTAqua research group (Nutrition, Genetics and Technology in Aquaculture/UFMG) were kept separated into two different tanks (useful volume of 3.6 m^3^ each) in clear water system. These animals were fed with vitamin C enriched feed as recommended by Mataveli et al. (2018). For the induction of triploidy, four females, out of the twenty-seven ones, that presented at the same time an enlarged and reddish genital papilla and cambered ventral region (“ready to spawn”) were chosen for reproduction induced with human chorionic gonadotropin (hCG) (Vetecor, Calier Laboratory, Spain) by a single dose of 1 IU/gram of female (Azevedo et al., 2021). After 24 hours, the four induced females were striped to obtain their oocytes that were fertilized by a pool of semen collected from three males, out of the fifteen males (the oocytes of each female were fertilized by 0.6–1 mL of semen). After dry fertilization, 10 mL of water from the egg incubation system was added. The total spawning volume of each female was divided into two batches (the same volume of eggs for each batch), the treatment and control batches. After 4 minutes the treatment batch was subjected to a 4-minute heat shock in water at 41°C (Pradeep et al., 2010). Then, the eggs were pooled in two groups, a pool of treated eggs and another of control eggs, and they were transferred to an artificial incubator (27 °C).

Before egg transfer, a random sample of 1.0 mL of eggs was obtained, fixed in Bouin's solution and used to measure the number of eggs in each pool (eggs ∙ mL^-1^ × volume of eggs in each group of treatment) and the same number of eggs were adjusted to be hatched (738 eggs per each group of treatment). The number of larvae was also measured after yolk sac absorption to determine the survival rate of hatched eggs. As only 160 larvae from the treated egg group hatched and survived, the same number of larvae (160) from the control group were kept for evaluation. The larvae were considered as the experimental units of the assay.

**Figure 1:**
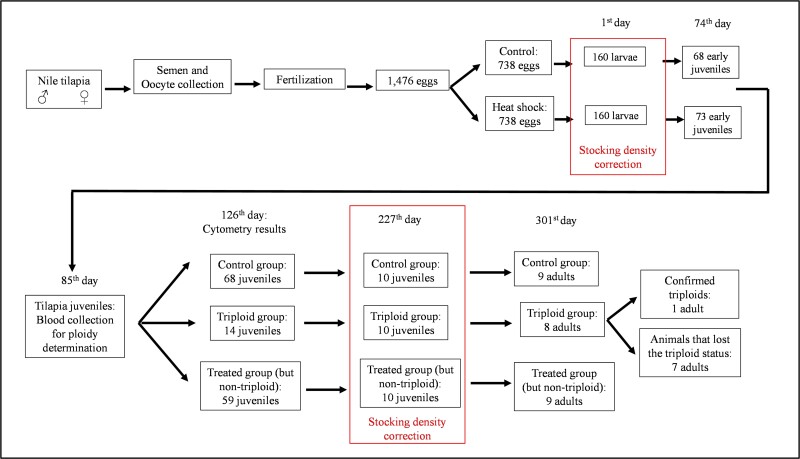
Experimental procedures schematic summary. Before egg incubation, the same number of eggs were adjusted to be hatched for control and heat shock groups (738 eggs for each group). Following yolk sac absorption, we counted the number of resultant larvae and equalized the stocking density of control group with the stocking density of the heat shock group (160 larvae per group, using the number of surviving larvae from heat shock group as reference). On the 74th day post yolk sac absorption, the early juveniles were recounted (68 and 73 juveniles from control and heat shock groups, respectively) and on the 85th day post yolk sac absorption, blood samples were collected for the first time to determine the ploidy by flow cytometry. Analysis of the DNA content histograms of the animals' blood cells indicated 14 triploids in the heat shock (treated) group. On 227th day post yolk sac absorption, a new stocking density adjustment was made according to the living triploids number (10 individuals). Therefore, 10 juveniles from the control group and others 10 juveniles from treated group (animals submitted to heat shock, but non-triploid) were randomly selected. At the end of the experiment, there were 8 animals from triploid group, 9 fish from control group and 9 tilapias from treated group. Blood samples were collected from all remain animals to evaluate the ploidy by flow cytometry in adult animals. In this second analysis, only one animal from triploid group was confirmed triploid.

The tilapias were kept in a closed production system until adulthood. The larviculture period was carried out in a water recirculation system in 30 L tanks (close to 1 larvae ∙ L^-1^), until the 74th day post yolk sac absorption and around 23 g of body weight. On that day, the early juveniles were recounted (68 and 73 juveniles from control and heat shock groups, respectively) and transferred to four 800 L tanks in biofloc system (two tanks per treatment, around 87.5 juveniles ∙ m^-3^). On 227th day post yolk sac absorption, a new stocking density adjustment was made according to the living triploids number (10 individuals). Therefore, 10 juveniles from the control group and others 10 juveniles from treated group (animals submitted to heat shock, but non-triploid) were randomly selected. Each group of 10 juveniles were kept in 800 L tank (3 tanks, around 12.5 juveniles ∙ m^-3^) in biofloc system until the end of the experiment. The overall experiment schematic summary is shown in Figure 1.

The juveniles were fed an initial ration that had 55% crude protein (Propescado-Nutriave Foods, Brazil), starting with a daily treat of 20% of biomass and a feeding frequency of eight times a day, according to Kubitza (2000). Granulometry, dietary protein level and feeding frequency were adjusted for tilapia growth according to the same author.

### Water quality

Water quality was evaluated twice a week. The parameters measured with digital equipment were temperature (°C), pH, and salinity (‰). Alkalinity (mg of CaCO_3_ ∙ L^-1^), total ammonia nitrogen (TAN), and nitric nitrogen (N-NO_2_^-^) were measured according to the protocols of APHA (2012), UNESCO (1983), and Bendschneider and Robinson (1952), respectively. Nitrogen nitrate (N-NO_3_^-^) was quantified by the methodology applied by Monteiro et al. (2003).

For the period in which the animals were raised in biofloc systems, commercial sugar (50% carbon) was used as an additional carbon source. The amount of sugar added to the system was calculated using a C:N ratio of 6:1 based on the TAN concentration according to Ebeling et al. (2006). Sedimentary solids (SS) were measured when the animals were in a biofloc system, with a 1 L Imhoff cone after settling for 15 min. The averages of the water quality parameters were within the recommended limits for tilapia (Table 1).

**Table 1:** Water quality parameters (means ± standard deviation) from the tanks in which tilapias hatched from eggs subjected or not to heat shock were cultivated.

**Parameters**	**Means ± standard deviation**	**Reference values**
Temperature (°C)	26.7 ± 3.10	27-32^(^1^)^
pH	6.6 ± 0.82	6-9^(^2^)^
Salinity (ppt)	1.5 ± 0.98	1-8^(^3^)^
Total ammonia nitrogen (mg∙L^-1^)	0.006 ± 0.03	<1^(1)^
Nitric nitrogen (mg∙L^-1^)	2.5 ± 0.64	<8^(1)^
Dissolved oxygen (mg∙L^-1^)	6.0 ± 1.21	>4^(2)^
Alkalinity (mg∙L^-1^)	39.2 ± 0.98	>20^(1)^
Settleable solids (mL∙L^-1^)	14.0 ± 8.61	2-40^(^4^)^
Nitrate (g∙L^-1^)	0.2 ± 0.06	<0.5^(^5^)^

^(1)^El-Sayed (2006); ^(2)^Wedemeyer (1996); ^(3)^Alvarenga et al. (2018); ^(4)^Avnimelech (2009); ^(5)^Monsees et al. (2017).

### Flow cytometry analysis

When the tilapias reached 85 days post-yolk sac absorption, they were anesthetized by immersion in water with eugenol solution (5% - 1 mL ∙ L^-1^ of water) and identified by Passive Integrated Transponder (PIT) tags. Then, ten μL of blood were collected and processed according to Alvarenga et al. (2020). The samples were stored in a refrigerator at 4ºC until analysis. Ploidy determination was performed as described by Herbst (2002). An estimate of the number of cells was obtained by counting erythrocytes/microliters. The ideal amount for the cytometry analysis is 10^5^ - 10^6^ cells/microliters. Blood samples were also collected at the end of the experiment (adult fish, 300 days) to recheck ploidy and assess chromosomal stability.

The cytometry was carried out at the Institute of Biosciences-UNESP, Botucatu, Brazil. Blood samples were centrifuged by 8 min to 300 g, the supernatant was discarded and added 1 ml of phosphate-biffered saline (PBS) (NaCl 0.8%, KCl 0.02%, Na_2_HPO_4_ 0.144%, KH_2_PO_4_ 0.024%) and blood cells were resuspended in 1 ml of PBS, and then centrifuged by 8 min to 300 g. The supernatant was discarded. Blood cells were resuspended in 1 mL of cell nuclei marking solution (1 mL of Triton X-100, 0.2 mg of RNase, 0.02 mg ∙ mL^-1^ propidio iodide (PI) in PBS). Data acquisition was performed in *FACSCanto^TM^ II* flow cytometer (BD Biosciences) with *FACSDiva* software (BD Biosciences). A rate of ten thousand events by sample was used. The gates were established based on size (FSC) and granularity (SSC) parameters and later PI fluorescence. Red blood sample cells of a known diploid tilapia were used as internal control.

The measurements of DNA content from control individuals (diploid) were compared to those that received heat treatment. The fish were classified as diploid (flow cytometry results equal to diploid control), aneuploid (histogram peak slightly displaced to the left compared to the diploid control histogram peak), triploid (histogram peak moved to the right compared to the diploid control histogram peak), as described by Zhang and Arai (1996). The results were analyzed using the Flowjo® software.

### Growth performance

To assess tilapia growth performance, body weight was measured on the 26th, 39th, 60th, 74th, 126th, 227th, 276th, and 301st day of age post yolk sac absorption with an analytical balance and the number of individuals was counted to obtain the survival rate (%). Tilapia were previously anesthetized by water immersion with eugenol solution (5% - 1 mL ∙ L^-1^ of water) (Ranzani-Paiva et al., 2013).

### Gonadal development

At 378 days of age post yolk sac absorption, the tilapias were euthanized, the gonads were removed and fragmented (~5 mm thick). These fragments were fixed by immersion in 5% glutaraldehyde and 0.05 M phosphate buffer (pH 7.3) and then embedded in methacrylate glycol. Sections were obtained using a Reichert Jung automatic microtome (NuBlock, Germany) and stained with 1% toluidine blue. Analyzes of these sections were performed on an Olympus IX70® light microscope to identify the presence of spermatozoa in males and vitellogenic oocytes in females.

### Statistical analysis

Prior to ploidy's first assessment, the comparison was performed between two groups: control (which did not receive treatment) and heat shock (which received heat shock treatment). After the first cytometry, the individuals were separated into three groups: control, treated group but non-triploid (which received heat shock but had no change in ploidy) and triploids. Individuals who after the first cytometry were classified as aneuploid were not used. For statistical analysis, the growth performance, water quality and reproductive variables were evaluated for normality (Shapiro-wilks test) and homoscedasticity (Bartlett test) using R software (R Core Team, 2020). The variables that met these ANOVA assumptions were submitted to it, followed by Duncan's test. For data that assumptions did not meet, the non-parametric Kruskal-Wallis test was used. Chi-square test was used to compare the survival rate. Fisher's exact test was used to compare the results of number of males and females between groups diploids, triploids and aneuploids. The level of significance was p < 0.05.

The experiment was conducted by the NGTAqua research group at the Aquaculture Laboratory of the Veterinary School (EV) of Federal University of Minas Gerais (UFMG), Brazil. All procedures were previously approved by the UFMG animal ethics committee under protocol number 215/2021.

## Results

The average body weight (g) and the survival rate (%) of animals from different growth stages are shown in Table 2 and Table 3. From the 1st day of age post yolk sac absorption (when the number of surviving larvae was equated) to the 126th, average body weight did not differ between groups. From the 126th to the 301st day of age post yolk sac absorption, the triploids had a higher average body weight. But at the final weighing (378 days of age post yolk sac absorption), body weight was not different between the groups. The survival rate did not differ between groups at any stage of life.

**Table 2:** Body weight (1^st^ quantile, median, and 3^rd^ quantile) and survival rate (proportion) of Nile tilapias from 26^th^ to 126^th^ day post yolk sac absorption, hatched from eggs subjected or not to heat shock for triploidy induction.

**Days after fertilization**	**Variables**	**Control group**			**Heat shockGroup**		
		**1Q**	**M**	**3Q**	**1Q**	**M**	**3Q**
26^th^ day	W (g)1	0.41	0.66	0.99	0.33	0.66	0.97
39^th^ day	W (g) ^1^	2.16	2.84	3.80	1.79	3.08	3.80
60^th^ day	W (g)^1^	8.06	11.26	13.30	7.96	12.48	14.98
74^th^ day	W (g)^1^	18.16	22.19	26.50	17.22	24.19	29.58
126^th^ day	W (g)^1^	45.00	65.00	80.00	45.00	57.50	90.00
		Rate				Rate	
	Sur (%)2	42.50			45.63		

Sur: Survival rate (from 26^th^ day to 126^th^ day after yolk sac absorption); W: average body weight; 1Q: 1^st^ quantile; M: median; 3Q: 3^rd^ quantile. ^1^Median do not differ by the Kruskal-Wallis test (p > .05); ^2^Values do not differ by Chi-square test (p >.05).

**Table 3:** Body weight mean, coefficient of variation (CV) and survival rate (proportion) of Nile tilapias from 126^th^ to 378^th^ day of age post yolk sac absorption, hatched from eggs subjected or not to heat shock for triploidy induction.

**Days after fertilization**	**Variables**	**Control group**	**Triploid group**	**Treated group**	**CV (%)**
126^th^ day	W (g)1	65.00^a^	55.00^a^	65.75^a^	42.28
227^th^ day	W (g)^1^	178.47^b^	241.25^a^	127.22^b^	32.44
276^th^ day	W (g)^1^	221.53^b^	347.50^a^	186.67^b^	34.63
301^st^ day	W (g)^1^	274.86^b^	397.50^a^	220.00^b^	35.13
	Sur (%)2	90	80	90	-
Days after fertilization	Variables	Control	Confirmed triploids	Animals that lost the triploid status	Treated non-triploids
378^th^ day	W (g)	326.82	940.00*	378.57	281.11

CV: Coefficient of variation; Sur: Survival rate (from 126^th^ day to 378^th^ day post yolk sac absorption); W: average body weight. ^1^Means followed by the same letter do not differ from each other by the Duncan test (p > .05); ^2^Values do not differ by Chi-square test (p >.05). *Only one animal had triploidy confirmed at the end of experiment.

The first analysis of the DNA content histograms of the animals' blood cells indicated 14 triploids in the heat shock (treated) group. The relative frequency of females and males according to the result of the first ploidy analysis were showed in Table 4. The sex ratio did not differ between groups.

**Table 4:** Relative frequency (%) and number (in parentheses) of males and females of Nile tilapia classified according to ploidy after heat shock for triploidy induction.

**Sex**	**Diploid1**	**Triploid^1^**	**Aneuploid^1^**
F	38.46% (10)	57.14% (8)	42.43% (14)
M	61.54% (16)	42.86% (6)	57.57% (19)
Total	100.00% (26)	100.00% (14)	100.00% (33)

F: Female; M: Male. ^1^Values do not differ by Fisher's exact test (p<0.05).

**Figure 2:**
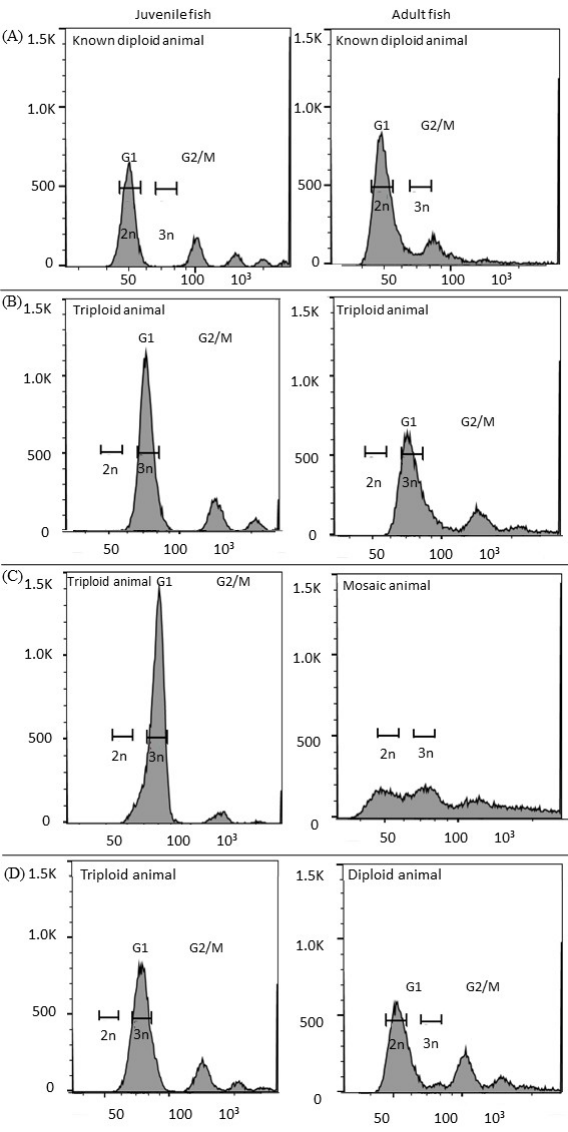
Representative histograms of DNA content of the animal’s blood cells from flow cytometry analysis. All cells with equal amounts of DNA content form a peak, one peak representing G1 and the other (twice the channel value) representing the G2/M phase of the cell cycle. The position of the G1 peak established the ploidy level of this material (Ochatt, 2006). With a known diploid animal, the position where the peak was determined and estimated the position of a triploid animal. In the first column we observed the results of the cytometry performed in juveniles and in the second column the analysis of the same tilapia in the adult phase. (A) Tilapia hatched from eggs not subjected to heat shock (control group). (B) Tilapia hatched from eggs subjected to heat shock, observed a shift of the peak to the right, demonstrating that the shock was efficient in inducing triploidy. However, in adult fish, the peak is wider, indicating greater chromosomal variability, probably due to aneuploidies. (C) Tilapia hatched from heat-shocked eggs, whose DNA content histograms indicated triploid status in the juvenile stage, but as adults it lost irregularly shaped sets of chromosomes, becoming mosaic. (D) Tilapia hatched from eggs subjected to heat shock, whose DNA content histograms indicated triploid status in the juvenile stage, but when adult it lost a set of chromosomes, becoming diploid.

At the end of the experiment, when the second analysis was made, there were 8 animals in the triploid group, 9 fish from control group and 9 tilapias from treated group (non-triploid). Only one individual out of 8 tilapias from triploid group (which corresponded to 12.5% of individuals) remained triploid. Thus, 87.5% of fish (7 individuals) lost sets of chromosomes. This loss was at least in the hematopoietic tissue that was analyzed and presents a high proliferation rate. From these 7 fish that lost triploid status as adults 2 individuals become aneuploid, 3 mosaic, and 2 diploids (Figure 2).

**Figure 3:**
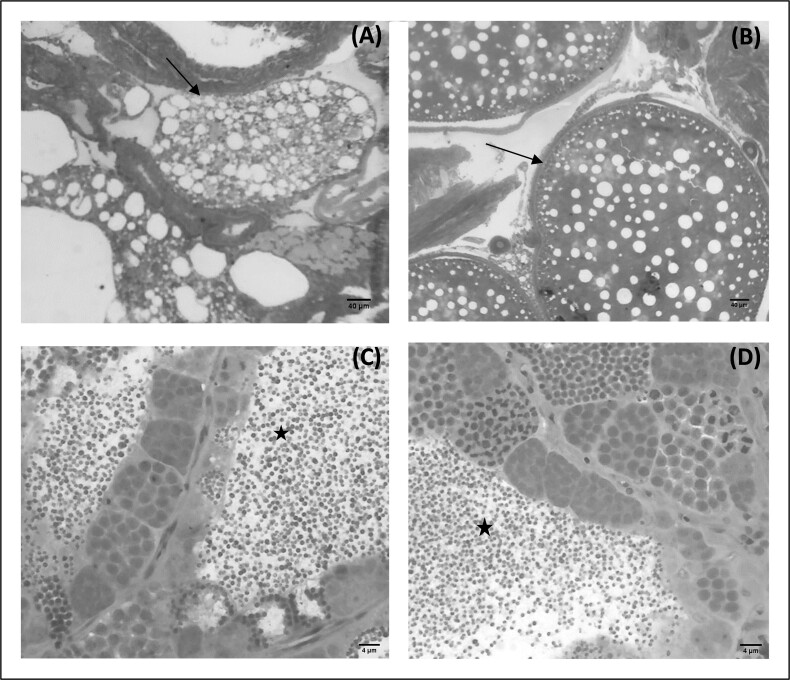
Photomicrograph of male and female Nile tilapia gonads. Observe an atretic follicle (A; arrow) of a triploid female and a vitellogenic follicle (B; arrow) of a control female. Spermatozoa founded in the lumen of seminiferous tubule (star) in the testis of a male that lost triploid status (C), and in diploid male (D).

The individual whose hematopoietic cells remained triploid was a female that had only atretic follicles (absence of vitellogenic follicles). The individuals who lost triploid status, all males had spermatozoa, 25% of the females had a predominance of atretic follicles, and 75% had a predominance of vitellogenic follicles. In the treated group, all males had spermatozoa, 66.7% of females had a predominance of vitellogenic follicles and 33.3%, a predominance of atretic follicles (Table 5 and Figure 3).

**Table 5:** Relative frequency (%) and number (in parenthesis) of the predominance of vitellogenic or atretic follicles in females and presence/absence of spermatozoa in males of Nile tilapia, hatched from eggs subjected or not to heat shock for triploidy induction.

**Sex**	**Parameter**	**Control**	**Confirmed triploids**	**Animals that lost the triploid status**	**Treated** **non-triploids**
Males	Presence of spermatozoa1	100% (4)^a^	0% (0)	100% (3)^a^	100% (6)^a^
	Absence of spermotozoa^1^	0% (0)^b^	0% (0)	0% (0)^b^	0% (0)^b^
Subtotal		100% (4)	100% (0)	100% (3)	100% (6)
Females	Predominance of vitellogenic oocytes^1^	100% (5)^a^	0% (0)^a^	75% (3)^a^	66.7% (2)^a^
	Predominance of Atretic follicle^1^	0% (0)^b^	100% (1)^a^	25% (1)^b^	33.3% (1)^b^
Subtotal		100% (5)	100% (1)	100% (4)	100% (3)
Total		(9)	(1)	(7)	(9)

^1^Values followed by the same letter in the column do not differ by Fisher's exact test (p > 0.05).

## Discussion

In this study, only one triploid individual maintained the same ploidy when Nile tilapias were analyzed in adult phase, and the others lost this status. As far as we know, an unprecedented situation for triploid fish. The results presented here raise the hypothesis that, at least in tissues with a high proliferation rate, such as the hematopoietic tissue that was analyzed and possibly in gonads, the loss of triploid status caused its development and probable the recovery of the reproductive capacity. In addition, the previously triploid and the other individuals equalized the average body weight.

From 126th to 301st day of age post yolk sac absorption in our study, triploid group (initially confirmed as triploid) performed better than animals not submitted to heat shock. Brämick et al. (1995) also observed a higher growth of Nile tilapia triploids (139 g) compared to diploid ones (81 g) at the end of 154 days of experiment. Even under different feeding regimes, a normal one (7 days/week of feeding) or a restricted one (5 days/week of feeding), Byamungu et al. (2001) obtained a higher growth for triploid blue tilapia juveniles compared to diploid ones, weighing around 70-110g. The literature shows that in other fish species, like yellowtail tetra *Astyanax altiparanae*, triploids had better growth rate, carcass yield and meat quality than diploid ones (Nascimento et al., 2017a, b). However, in our study, the triploid group weight performance equaled the control one when adulthood was analyzed, this final result must be associated with the chromosomes loss observed for most triploid individuals. This event indicates that the loss of chromosomes can occur in advanced tilapia phases of development and has not been observed in studies that analyzed the ploidy in early stages of development.

The survival of triploid individuals is comparable to diploid individuals from post-larval stages (Pechsiri and Yakupitiyage, 2005; Fraser et al., 2012). Byamungu et al. (2001) reported a survival rate of 65.3% and 60.7% for diploid and triploid *Oreochromis aureus*, respectively. In the present study, we did not obtain statistical differences regarding the survival of triploid and diploid animals, but high mortality in the larval phase was observed in triploids. A high mortality was also observed in larvae hatched from eggs treated with heat shock when it was not yet known whether they were triploid or not.

The sex ratio did not differ between triploid, treated and control groups. Likewise, Chang et al. (1991) reported no difference between the sex ratio in diploids and triploids of *Oreochromis aureus*. However, the number of triploid tilapias obtained in this induction was small (14 out of 73 surviving juveniles from the heat shock group) and the male/female ratio equality may have been due to the small sample size of surviving triploid tilapia. Brämick et al. (1995) observed that the overall sex ratio (males:females) was balanced in triploid tilapia too and these authors evaluated a larger number, 63 triploid. However, Pradeep et al. (2012) observed that fish subjected to heat shock at 41 °C produced an asymmetry to males, 84.1% of males, in relation to control (28 °C), 50.9% of males, and cold shock treatment (9° C), 54.7% of males. The same authors in a study published in 2013 obtained the same asymmetry (Pradeep et al., 2013)

In our study, the ploidy of tilapias in the initial phase of life and in the adult phase was analyzed, and it was observed that most of the triploid animals when young were no longer triploid as adults. In this sense, the efficiency of adult triploid production is even lower than the estimated efficiency of juvenile tilapia. Chromosomal loss in polyploids is frequent and occurs to a much greater extent than in diploids (Comai, 2005). Zhang et al. (2010, 2014) founded that oysters with increased ploidy in their cells tended to have higher percentages of aneuploidy, concluding that the general polyploidy condition provides the substrate for subsequent loss of chromosomes. For oysters, chromosomal loss is not limited to aneuploidy, but includes the loss of a complete set of chromosomes to become mosaics through a process called reversal (Zhang et al., 2010). Some findings suggested that the reversal existed in both tetraploid and triploid oyster and chromosomal agglomeration is the mechanism for chromosomal loss in polyploid. Cells that suffer reversal continually eliminate chromosomes until a stable (genetically healthy) state is established. It is assumed that the mitotic chromosome segregation cells are affected in cells with abnormal chromosome, resulting in daughter cells with unusual chromosomal constitutions (Zhang et al., 2014).

For vertebrate animals, chromosomal instability was reported in cattle clones. Two clones were evaluated at various times up to 20 months of age and the incidence of abnormal lymphocytes since they derived from different donor cell cultures with high abnormal cell incidences. The incidence of abnormal lymphocytes in clones remained stable, indicating a transient event of chromosomally abnormal nuclei found in the cloned animals. These results show that most phenotypically normal clones have normal chromosomal composition, but the instability of the number of chromosomes can occur (Hanada et al., 2005).

Chromosomal loss has not been reported in triploid fish within the same generation yet, however, in most triploid trials, the number of chromosomes in the cells is quantified in early stages of development and there is no triploidy status monitoring throughout developmental stages. Benfey (2016) also hypothesized chromosomal instability in fish and pointed out that a possible cause of the occasional occurrence of triploid individuals is either the mixing of ploidy levels between different cells (i.e., emergence of animals in mosaic) or the reversion to a state fully diploid. In the review on triploidy effectiveness as a management tool for reproductive control of farmed fish, the author mentioned that mosaic individuals have been reported as a result of triploid induction of Atlantic salmon, but that may be erroneous results due to errors in erythrocyte size measurements, and therefore the instability of triploids has not yet been reported in fish. Two theories for the complete triploid reversal were presented by Benfey (2016). One would be the loss of one or some chromosomes at a time, presumably through errors in cell divisions. The other theory is the loss of an entire haploid set chromosomes defined in a single cell division event.

In our study, during the cytometry analysis of adult animals, an abnormal amount of DNA was observed in the cells. Sometimes the triploidy induction results in mosaic individuals, where the level of ploidy varies between tissues (Teplitz et al., 1994; Arai, 2001). The presence of mosaic individuals was observed in other fish species after triploidy induction (Ewing et al., 1991; Teplitz et al., 1994; Goudie et al., 1995). When this occurs, it is important to verify whether the precursors of the germ cells are triploid, since the interest of triploidy is to be a direct method to guarantee the sterility of the animal. Sterility would not be guaranteed if mosaicism affected gametes as it affects other cells (Piferrer et al., 2009). The individual who remained triploid have only atresic follicles (instead of vitellogenic follicles), which may indicate that animals that reach triploid adult stage can be considered sterile. Benfey (2016) comments that there are several reports of triploid females that occasionally spawn mature oocytes, showing that perhaps the loss of chromosomes has occurred in these triploid individuals. Johnstone et al. (1991) estimated that 0.1% of the triploid Atlantic salmon female population ovulate and when fertilized with sperm from diploid males, produce aneuploid embryos that die early in development, the same was reported by Benfey (1996) with females of *Salvelinus fontinalis*.

In the present study, most adult animals lost triploid status, indicating the possible unfeasibility of using triploidy on a commercial scale for tilapiculture. Triploidy induction is well established for salmonids and already has commercial application, but in all current studies the animals were tested in the youth phase (average weight of 5 grams) (Leclercq et al., 2011; Taylor et al., 2011, 2012, 2013, 2014, 2015). For tilapia, triploidy induction is only at the level of laboratory experimentation and there are no reports of large-scale use of triploid individuals. Brämick et al. (1995) evaluated that of all 2,139 fish submitted to thermal shock to obtain triploidy, only 63 were triploid (= 3%). One hypothesis raise is that in salmonids, triploidy occurs spontaneously in the natural habitat (Cuellar and Uyeno, 1972; Thorgaard and Gall, 1979; Allendorf and Thorgaard, 1984; Leclercq et al., 2011; Glover et al., 2015; Jørgensen et al., 2018) probably facilitating the application of the technique, the survival of a large number of triploid adult salmonids and its feasibility, and for tilapia there are no reports for such a case.

## Conclusion

The results presented here demonstrate that there is still much to be investigated about chromosomal manipulation as a sterility tool for tilapia and other fish. Mainly pointing out the risks in the production of fish considered as triploids, in which their ploidy condition was evaluated only in the early stages of life. Reversal can be a problem for biological control and, depending on its extent, possibly for commercial aquaculture. The elimination of a chromosome set during triploid somatogenesis can have an evolutionary meaning of restoring the genome balance near to normal state. This loss in tissues with a high proliferation rate, such as in gonads, can result in diploid germ cells, which can lead to fertile haploid gametes formation. In other words, the reproductive capacity can be recovered in animals originally considered sterile and growth performance becomes similar to those of diploid animals. It is important to reassess the feasibility of using triploids as an option to produce Nile tilapia or even as a research model. Our results suggest that due to genomic instability caused by polyploidization, the generating of 3N Nile tilapias may have low efficiency.
